# 1096. Fully Vaccinated Individuals with Immunocompromised Conditions (IC) are Still at Increased Risk of Severe COVID-19 Outcomes From the Omicron Variant: Initial Results From INFORM, a Retrospective Health Database Observational Study in England

**DOI:** 10.1093/ofid/ofad500.069

**Published:** 2023-11-27

**Authors:** Sabada Dube, Yi Lu, Richard McNulty, Sophie Graham, Sofie Arnetorp, Nahila Justo, Renata Yokota, Kathryn Evans, Sudhir Venkatesan, Mark Yates, Sylvia Taylor, Jennifer Quint, Rachael A Evans

**Affiliations:** Medical Evidence, Vaccines and Immune Therapies Unit, AstraZeneca, Cambridge, UK;, Cambridge, England, United Kingdom; Real-World Evidence, Data Analytics, Evidera, London, UK, London, England, United Kingdom; Medical Affairs, Vaccines and Immune Therapies Unit, AstraZeneca, Cambridge, UK, Cambridge, England, United Kingdom; Real-World Evidence, Data Analytics, Evidera, London, UK, London, England, United Kingdom; Health Economics and Payer Evidence, BioPharmaceuticals R&D, AstraZeneca, Gothenburg, Sweden, Gothenburg, Vastra Gotaland, Sweden; Real-World Evidence, Data Analytics, Evidera, Stockholm, Sweden and Department of Neurobiology, Care Science and Society, Karolinska Institute, Stockholm, Sweden, Stockholm, Sodermanlands Lan, Sweden; P95, Leuven, Belgium, Dilbeek, Luxembourg, Belgium; Real-World Evidence, Data Analytics, Evidera, Waltham, MA, USA, Waltham, Massachusetts; Medical and Payer Evidence Statistics, BioPharmaceutical Medical, AstraZeneca, Cambridge, UK, Cambridge, England, United Kingdom; Real-World Evidence, Data Analytics, Evidera, London, UK, London, England, United Kingdom; Medical Evidence, Vaccines and Immune Therapies Unit, AstraZeneca, Cambridge, UK, Cambridge, England, United Kingdom; National Heart and Lung Institute, Imperial College London, London, UK, Cambridge, England, United Kingdom; University of Leicester, Leicester, England, United Kingdom

## Abstract

**Background:**

UK vaccination programs have reduced COVID-19–related hospitalizations and deaths in the overall population, yet vaccinated individuals with immunocompromised conditions (IC) are still at high risk of severe COVID-19 outcomes. Contemporary evidence on severe outcomes in vaccinated individuals with IC is needed, particularly in the post-pandemic omicron period. Initial results from INFORM, an observational retrospective cohort study describing COVID-19 health burden in individuals with and without IC vaccinated with ≥ 3 doses of a COVID-19 vaccine in England, are presented.

**Methods:**

Data from primary and secondary care linked to COVID-19 surveillance, vaccination records, primary care dispensations, and mortality, were accessed via National Health Service (NHS) database (**Figure 1**). The study period was Jan 1, 2022–Dec 31, 2022. Baseline characteristics were identified during the period Jan 1, 2017–Dec 31, 2021. Incidence rates (IR) are presented per 100 person-years (PY).

**Figure 1.**
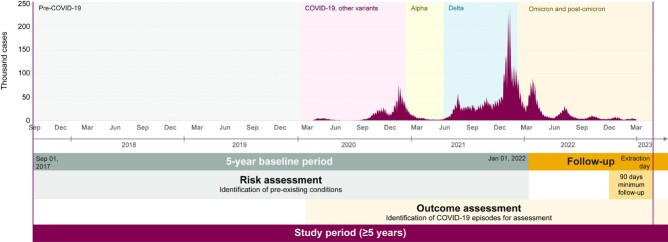
Study design: observational retrospective database study

**Results:**

Almost 90% of individuals with IC received ≥ 3 vaccine doses compared with 60% in the overall population aged ≥ 12 years (**Table 1**). Among individuals receiving ≥ 3 vaccine doses, COVID-19 hospitalization IRs were 0.92 (95% confidence interval [CI]: 0.89–0.95) in individuals with IC, compared with 0.22 (95% CI: 0.21–0.23) per 100 PY in the overall population. When analyzed by IC type, IRs were higher than the total vaccinated population across all IC subgroups (RR = 2.5–21) (**Table 2**). COVID-19 hospitalization IRs in patients with hematological malignancies currently under treatment were 4.65 (95% CI: 4.42–4.88) and those with organ transplants were 2.95 per 100 PY (95% CI: 2.7–3.2) (**Table 2**). Mortality IRs per 100 PY were also consistently higher across all IC groups.

**Figure:**
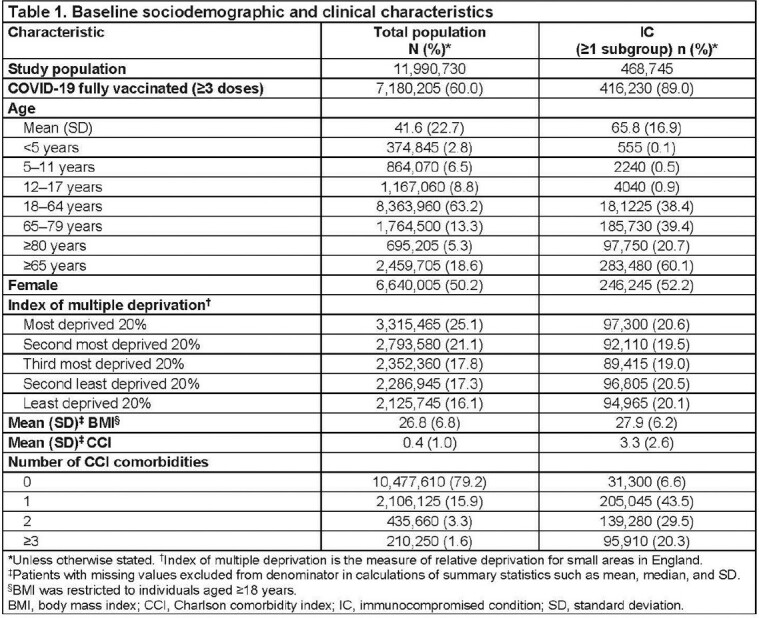

**Figure:**
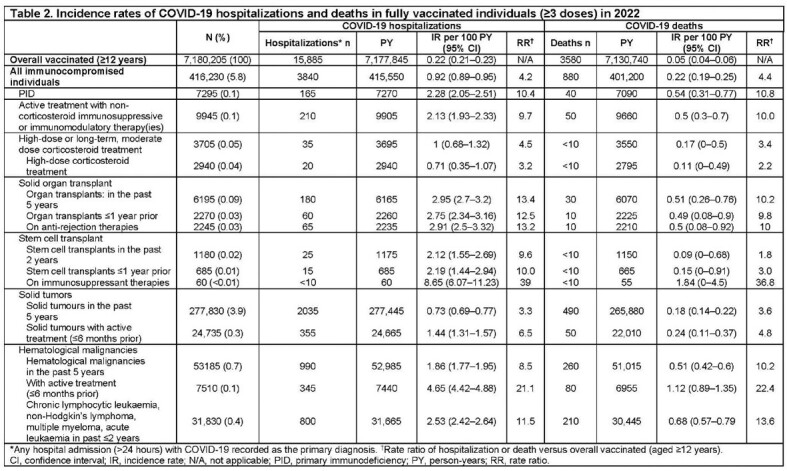

**Conclusion:**

Within a population vaccinated with ≥ 3 doses, individuals with IC had a higher risk of COVID-19 hospitalization and COVID-19 death compared with the overall population. This risk of severe COVID-19 outcomes was elevated across all IC groups, regardless of past or current treatment or procedure despite being vaccinated.

**Disclosures:**

**Sabada Dube, PhD**, AstraZeneca: Employee **Yi Lu, PhD**, Evidera: Employee **Richard McNulty, MD**, AstraZeneca: Employee **Sophie Graham, MSc**, Evidera: Employee **Sofie Arnetorp, MS**, AstraZeneca: Employee **Nahila Justo, PhD, MBA**, Evidera: Employee|Karolinska Institute: Employee **Renata Yokota, PhD**, AstraZeneca: Employee **Kathryn Evans, MPH**, Evidera: Employee **Sudhir Venkatesan, MPH, PhD**, AstraZeneca: Employee **Mark Yates, PhD**, Evidera: Employee **Sylvia Taylor, PhD, MPH, MBA**, AstraZeneca: Stocks/Bonds **Jennifer Quint, PhD**, AstraZeneca: Grant/Research Support|Evidera: Grant/Research Support|GlaxoSmithKline: Grant/Research Support|Insmed: Grant/Research Support **Rachael A. Evans, PhD FRCP**, AstraZeneca: Advisor/Consultant|Boehringer: Advisor/Consultant|Evidera: Advisor/Consultant

